# QUBO formulations for training machine learning models

**DOI:** 10.1038/s41598-021-89461-4

**Published:** 2021-05-11

**Authors:** Prasanna Date, Davis Arthur, Lauren Pusey-Nazzaro

**Affiliations:** 1 Oak Ridge National Laboratory, Oak Ridge, TN 37830 USA; 2 Auburn University, Auburn, AL 36849 USA; 3 Washington University in St. Louis, St. Louis, MO, 63130 USA

**Keywords:** Computer science, Information theory and computation

## Abstract

Training machine learning models on classical computers is usually a time and compute intensive process. With Moore’s law nearing its inevitable end and an ever-increasing demand for large-scale data analysis using machine learning, we must leverage non-conventional computing paradigms like quantum computing to train machine learning models efficiently. Adiabatic quantum computers can approximately solve NP-hard problems, such as the quadratic unconstrained binary optimization (QUBO), faster than classical computers. Since many machine learning problems are also NP-hard, we believe adiabatic quantum computers might be instrumental in training machine learning models efficiently in the post Moore’s law era. In order to solve problems on adiabatic quantum computers, they must be formulated as QUBO problems, which is very challenging. In this paper, we formulate the training problems of three machine learning models—linear regression, support vector machine (SVM) and balanced k-means clustering—as QUBO problems, making them conducive to be trained on adiabatic quantum computers. We also analyze the computational complexities of our formulations and compare them to corresponding state-of-the-art classical approaches. We show that the time and space complexities of our formulations are better (in case of SVM and balanced k-means clustering) or equivalent (in case of linear regression) to their classical counterparts.

## Introduction

The importance of machine learning algorithms in scientific advancement cannot be understated. Machine learning algorithms have given us great predictive power in medical science^1^, economics^2^, agriculture^3^ etc. These algorithms can only be implemented and deployed after they have been trained—a process that requires tuning the model parameters of a machine learning model in order to extract meaningful information from data. Training a machine learning model is a time and compute intensive process usually. In such situations, one is often forced to make a trade-off between the accuracy of a trained model and the training time. With the looming end of Moore’s law and rapidly increasing demand for large-scale data analysis using machine learning, there is a dire need to explore the applicability of non-conventional computing paradigms like quantum computing to accelerate the training of machine learning models.

Quantum computers are known to bypass classically-difficult computations by performing operations on high-dimensional tensor product spaces^4^. To this extent, we believe that machine learning problems, which often require such manipulation of high-dimensional data sets, can be posed in a manner conducive to efficient quantum computation. Quantum computers have been shown to yield approximate solutions to NP-hard problems, such as the quadratic unconstrained binary optimization (QUBO) problem^5^, graph clustering problem^6^, protein folding problem^7^ etc. In addition to these results, demonstration of quantum supremacy by Google^8^ has led us to believe that quantum computers might offer speedup in a much wider range of problems such as accelerating training of machine learning models.

To this extent, the principal contributions of our work are:

1. We formulate the training problems of three machine learning models—linear regression, support vector machine (SVM) and balanced *k*-means clustering—as QUBO problems so that they can be trained on adiabatic quantum computers.
2. For the aforementioned models, we provide a theoretical comparison between state-of-the-art classical training algorithms and our formulations that are conducive to being trained on adiabatic quantum computers. We observe that the time and space complexities of our formulations are better in case of SVM and balanced *k*-means clustering, and equivalent in case of linear regression, to their classical counterparts.

Our formulations provide a promising outlook for training such machine learning models on adiabatic quantum computers. In the future, larger and more robust quantum computers are sought to abate the limitations of current machines and potentially allow machine learning models to be trained faster and more reliably.

## Related work

Quantum machine learning algorithms have been proposed for both universal and adiabatic quantum computers. We briefly review a handful of such algorithms that leverage universal quantum computers here. Relevant algorithms leveraging adiabatic quantum computers have been reviewed in the subsequent sections. Quantum machine learning algorithms, and in general, all quantum algorithms will greatly benefit from optimal design of quantum circuits^9,10^, optimized quantum states^11^, quantum memory^12^, improved quantum coherence times^13^ and quantum error correction^14^. Today’s quantum machine learning algorithms are catered towards quantum computers in the noisy intermediate-scale quantum (NISQ) era. Results presented in this paper are part of our ongoing work to accelerate training of machine learning models using quantum computers^15–17^.

Farhi et al. proposed the Quantum Approximate Optimization Algorithm (QAOA), which produces approximate solutions for combinatorial optimization problems^18–20^, is computationally universal^21^, and has been used to train unsupervised machine learning models^22^. Farhi and Neven also proposed quantum neural networks where a sequence of parameter dependent unitary transformations act on classical or quantum input data and produce classification predictions on the output qubits^23^. Gyongyosi and Imre proposed training optimizations for such gate-based quantum neural network models^24^. Benedetti et al.^25^ proposed the use of the variational quantum eigensolver (VQE) algorithm in conjunction with parameterized quantum circuits as quantum machine learning models. QAOA and VQE based quantum machine learning models are widely used in the literature.

## Adiabatic quantum computers

The adiabatic theorem states that a quantum physical system remains in its instantaneous eigenstate under a slowly acting perturbation if there is a gap between its eigenvalue and the rest of the Hamiltonian’s spectrum^26^. Adiabatic quantum computers leverage the adiabatic theorem to perform computation^27^. Specifically, starting with the global minimum of a simple Hamiltonian, they homotopically connect it to the global minimum of the problem of interest^28^. The D-Wave adiabatic quantum computers, for instance, are adept at approximately solving the quadratic unconstrained binary optimization (QUBO) problem, which is stated as follows:

1 $$\begin{aligned} \min _{z \in {\mathbb {B}}^M} \ z^T A z + z^T b \end{aligned}$$

where, *M* is a natural number; ${\mathbb {B}} = \{0, 1\}$ is the set of binary numbers; $z \in {\mathbb {B}}^M$ is the binary decision vector; $A \in {\mathbb {R}}^{M \times M}$ is the real-valued $M \times M$ QUBO matrix; and, $b \in {\mathbb {R}}^M$ is the real-valued *M*-dimensional QUBO vector.

## Notation

We use the following notation throughout this paper:

- ${\mathbb {R}}$, ${\mathbb {N}}$, ${\mathbb {B}}$: Set of real numbers, natural numbers and binary numbers (${\mathbb {B}} = \{0, 1\}$).
- *N*: Number of data points (number of rows) in the training data set, $N \in {\mathbb {N}}$.
- *d*: Number of features (number of columns) in the training data set, $d \in {\mathbb {N}}$.
- *X*: Training data set, usually $X \in {\mathbb {R}}^{N \times d}$, i.e. *X* contains *N* data points along its rows, and each data point is a *d*-dimensional row vector.
- *Y*: Regression labels of the training data set in case of regression ($Y \in {\mathbb {R}}^N$); classification labels of the training data set in case of support vector machine ($Y \in {\mathbb {B}}^N$).

## Linear regression

### Background

Linear regression is one of the oldest statistical machine learning techniques that is used in a wide range of applications, such as scientific research^29^, business^30^ and weather forecasting^31^. Linear regression models the relationship between a dependent variable and one or more independent variables.

Adiabatic quantum computing approaches have been proposed in the literature for solving the linear regression problem (Eq. 2). Borle et al. propose a quantum annealing approach for the linear least squares problem^32^. Chang et al. present a quantum annealing approach for solving polynomial systems of equations using least squares^33^. Chang et al. propose a method for solving polynomial equations using quantum annealing and discuss its application to linear regression^34^. While these approaches can only find positive real-valued regression weights, our formulation finds both positive and negative real-valued regression weights.

Here, we denote $X \in {\mathbb {R}}^{N \times (d+1)}$ as the augmented regression training data matrix, where we have augmented each row of the original $X \in {\mathbb {R}}^{N \times d}$ with unity for the sake of mathematical convenience. The regression training labels are denoted by $Y \in {\mathbb {R}}^{N}$, and the regression weights are denoted by $w \in {\mathbb {R}}^{d+1}$. Given *X* and *Y*, training a linear regression model can be stated as follows:

2 $$\begin{aligned} \min _{w \in {\mathbb {R}}^{d+1}} \ E(w)&= || Xw - Y ||^2 \end{aligned}$$

**Figure 1:**
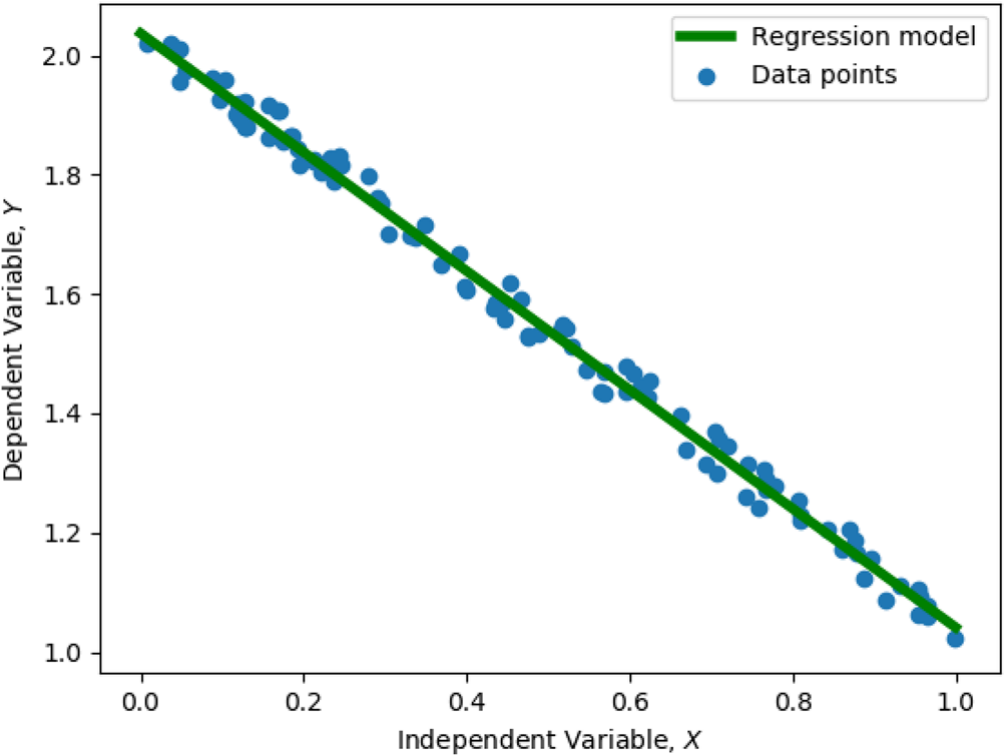
Fitting a linear regression model (green line) to data (blue dots).

Here, *E*(*w*) is the Euclidean error function. With reference to Fig. 1, the blue dots represent the data points *X* and *Y*, and the green line, characterized by the weights *w*, is the regression hyperplane which fits the data. The regression problem has an analytical solution, given by

3 $$\begin{aligned} w = (X^T X)^{-1} X^T Y \end{aligned}$$

If $(X^T X)^{-1}$ does not exist, the pseudo inverse is computed. Time complexity of linear regression is known to be ${\mathcal {O}}(N d^2)$.

### QUBO formulation

We start by rewriting Problem (2) as:

4 $$\begin{aligned} \min _{w \in {\mathbb {R}}^{d+1}} \ E(w)&= w^T X^T X w - 2 w^T X^T Y + Y^T Y \end{aligned}$$

Next, we introduce a *K*-dimensional precision vector $P = [p_1, p_2, \ldots , p_K]^T$. Each entry in *P* can be an integral power of 2, and can be both positive or negative. We also introduce a *K*-dimensional vector $\hat{w_i} \in {\mathbb {B}}^K$ with binary coefficients, such that the inner product $\hat{w_i}^T P$ yields a scalar $w_i \in {\mathbb {R}}$. This scalar $w_i$ represents the $i{\text {th}}$ entry in our weight vector, where $1 \le i \le (d+1)$. The entries of *P* must be sorted, for instance $P = \left[ -2, -1, -\frac{1}{2}, \frac{1}{2}, 1, 2, \right] ^T$. ${\hat{w}}_{ik}$ can be thought of as a binary decision variable that selects or ignores entries in *P* depending on whether its value is 1 or 0 respectively. With this formulation, we can have up to $2^K$ unique values for each $w_i$ when *P* contains only positive values for instance. However, if *P* contains negative values as well, then the number of unique attainable values for each $w_{i}$ might be less than $2^K$. For example, if $P = [-1, -\frac{1}{2}, \frac{1}{2}, 1]$, then only the following seven distinct values can be attained: $\{-\frac{3}{2}, -1, -\frac{1}{2}, 0, \frac{1}{2}, 1, \frac{3}{2}\}$.

Now, let us define the binary vector ${\hat{w}} \in {\mathbb {B}}^{K(d+1)}$, such that

5 $$\begin{aligned} {\hat{w}}&= [{\hat{w}}_{11} \ \ldots \ {\hat{w}}_{1K} \ {\hat{w}}_{21} \ \ldots \ {\hat{w}}_{2K} \ \ldots \ {\hat{w}}_{(d+1)1} \ \ldots \ {\hat{w}}_{(d+1)K}]^T \end{aligned}$$

Similarly, we can define a precision matrix (${\mathcal {P}}$) as follows:

6 $$\begin{aligned} {\mathcal {P}} = I_{d+1} \otimes P^T \end{aligned}$$

where $I_{d+1}$ represents the $(d+1)$-dimensional identity matrix, and $\otimes$ represents the Kronecker product. Note that ${\mathcal {P}}$ has the dimensions $(d+1) \times K(d+1)$. We can now recover our original weight vector by observing that:

7 $$\begin{aligned} w&= {\mathcal {P}} {\hat{w}} \end{aligned}$$

We have thus represented our weight vector (to finite precision) in terms of the precision matrix ${\mathcal {P}}$ and the binary vector ${\hat{w}} \in {\mathbb {B}}^{K(d+1)}$. We are now able to pose the minimization problem of Eq. (4) as an equivalent QUBO problem. Let us substitute the expression we obtained for the weight vector *w* in terms of ${\mathcal {P}}$ and ${{\hat{w}}}$ into Eq. (4), which yields:

8 $$\begin{aligned} \min _{{\hat{w}} \in {\mathbb {B}}^{(d+1)K}} \ E({\hat{w}})&= {\hat{w}}^T {\mathcal {P}}^T X^T X {\mathcal {P}} {\hat{w}} - 2 {\hat{w}}^T {\mathcal {P}}^T X^T Y \end{aligned}$$

Note that we have neglected the term $Y^TY$ because it is a constant scalar and does not affect the optimal solution to this unconstrained optimization problem. Observe that Eq. (8) now has the form of a QUBO problem, as desired. Hence, we can solve this optimization problem using an adiabatic quantum computer.

### Computational complexity

The regression problem (Problem 2) has ${\mathcal {O}}(N d)$ data (*X* and *Y*) and ${\mathcal {O}}(d)$ weights (*w*), which is the same for Problem (8). We introduced *K* binary variables for each of the $d+1$ weights when converting Problem (2) to Problem (8). So, we have ${\mathcal {O}}(d K)$ variables in Eq. (8), which translates to quadratic qubit footprint (${\mathcal {O}}(K^2 d^2)$) using an efficient embedding algorithm such as the one proposed by Date et al.^5^ Embedding is the process of mapping logical QUBO variables to qubits on the hardware, and is challenging because inter-qubit connectivity on the hardware is extremely limited. So, the space complexity of our approach is ${\mathcal {O}}(K^2 d^2)$.

Solving the regression problem takes ${\mathcal {O}}(N d^2)$ time classically. We analyze the time complexity of our approach in three parts: (i) Time taken to convert the regression problem into QUBO problem; (ii) Time taken to embed the QUBO problem onto the hardware; and (iii) Time taken to perform quantum annealing. From Eq. (8), we can infer that the conversion takes ${\mathcal {O}}(N d^2 K^2)$ time. Since we have ${\mathcal {O}}(dK)$ variables in the QUBO formulation, embedding can be done in ${\mathcal {O}}(d^2 K^2)$ time using the embedding algorithm proposed by Date et al.^5^. While the theoretical time complexity of quantum annealing to obtain an exact solution is known to be exponential (${\mathcal {O}}(e^{\sqrt{d}})$)^35^, a more realistic estimate of the running time can be made by using measures such as ST99 and ST99(OPT)^36^, which give the expected number of iterations to reach a certain level of optimality with $99\%$ certainty. Quantum annealing is known to perform well on problems where the energy barriers between local optima are tall and narrow because such an energy landscape is more conducive to quantum tunneling. In order to estimate ST99 and ST99(OPT) for our approach, details on specific instances of the regression problem are required. It remains out of the scope of this paper to estimate ST99 and ST99(OPT) for generic QUBO formulation of the regression problem.

Having said that, we would like to shed some light on the quantum annealing running times observed in practice. An adiabatic quantum computer can only accommodate finite-sized problems—for example, D-Wave 2000Q can accommodate problems having 64 or fewer binary variables requiring all-to-all connectivity^5^. For problems within this range, a constant annealing time and a constant number of repetitions seem to work well in practice. So, the total time to convert and solve a linear regression problem on adiabatic quantum computer would be ${\mathcal {O}}(N d^2 K^2)$.

It may seem that this running time is worse than its classical counterpart. However, the above analysis assumes that *K* is variable. On classical computers, the precision is fixed, for example, 32-bit or 64-bit precision. We can analogously fix the precision for quantum computers, and interpret *K* as a constant. The resulting qubit footprint would be ${\mathcal {O}}(d^2)$, and the time complexity would be ${\mathcal {O}}(N d^2)$, which is equivalent to the classical approach.

## Support vector machine (SVM)

### Background

**Figure 2:**
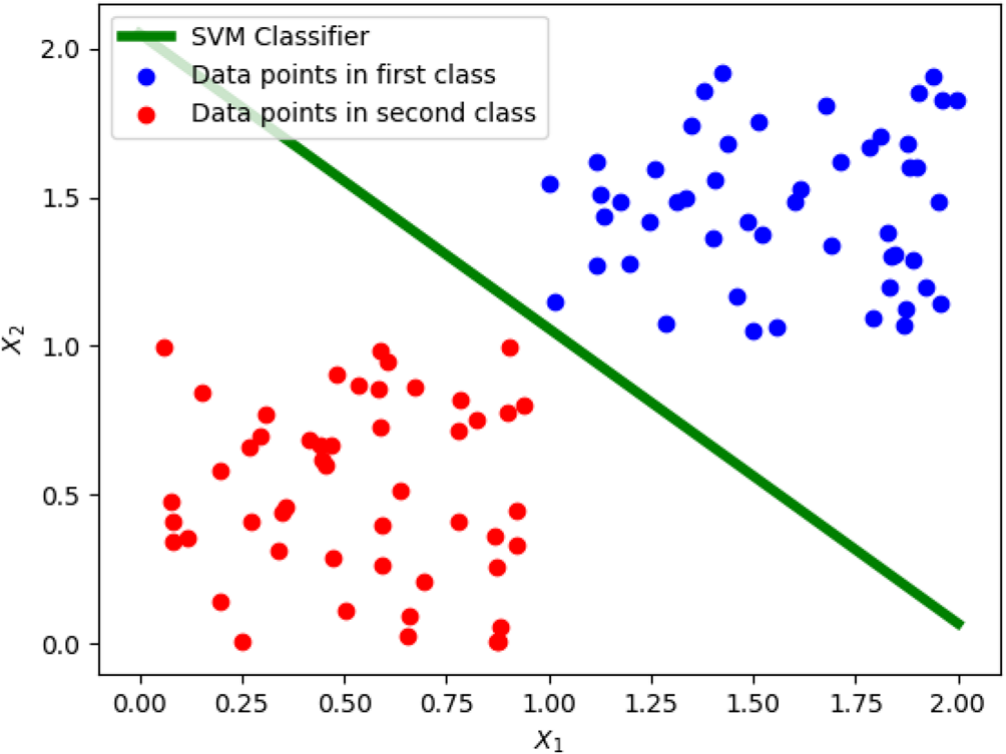
SVM model (green line) correctly classifying training data (red and blue dots).

Support vector machine (SVM) is a powerful supervised machine learning model that produces robust classifiers as shown in Fig. 2. The classifier produced by SVM maximizes its distance from the classes of the data points. Although SVM was meant for binary classification originally, several variants of SVM have been proposed over the years that allow multi-class classification^37,38^. SVM has wide ranging applications in multimedia (vision, text, speech etc.)^39^, biology^40^, and chemistry^41^, among many other scientific disciplines.

Some quantum approaches for training SVM using adiabatic quantum computers have been proposed in the literature. Ahmed proposes a formulation for quantum SVM that runs on noisy intermediate-scale quantum (NISQ) processors^42^. Welsh et al. propose a formulation of SVM for the D-Wave quantum computers^43^. Our findings improve upon their formulation, allowing for real-valued learning parameters up to a certain precision.

Given training data $X \in {\mathbb {R}}^{N \times d}$ and training labels $Y \in \{-1, +1\}^N$, we would like to find a classifier (determined by weights, $w \in {\mathbb {R}}^d$, and bias, $b \in {\mathbb {R}}$), that separates the training data. Formally, training SVM is expressed as:

9 $$\begin{aligned}&\min _{w, \ b} \ \frac{1}{2} ||w||^2 \nonumber \\&\quad \text {subject to:} \quad&y_i (w^T x_i + b) \ge 1 \qquad \forall i = 1, \ 2, \ \ldots , \ N \end{aligned}$$

Note that $x_i$ is the $i{\text {th}}$ row vector in *X* and $y_i$ is the $i{\text {th}}$ element in *Y*. The objective function is convex because its Hessian matrix is positive semidefinite. Furthermore, since the constraints are linear, they are convex as well, which makes Problem (9) a quadratic programming problem. To solve Problem (9), we first compute the Lagrangian as follows:

10 $$\begin{aligned} {\mathcal {L}}(w, b, \lambda ) = \frac{1}{2} ||w||^2 - \sum _{i = 1}^{N} \lambda _i \left[ y_i (w^T x_i + b) - 1 \right] \end{aligned}$$

where, $\lambda$ is the vector containing all the Lagrangian multipliers, i.e. $\lambda = [\lambda _1 \ \lambda _2 \ \cdots \ \lambda _N]^T$, with $\lambda _i \ge 0 \quad \forall i$. The non-zero Lagrangian multipliers in the final solution correspond to the support vectors and determine the hyperplanes $H_1$ and $H_2$ in Fig. 2. The Lagrangian dual problem (Eq. 10) is solved in ${\mathcal {O}}(N^3)$ time on classical computers by applying the Karush-Kuhn-Tucker (KKT) conditions^44,45^. As part of the KKT conditions, we set the gradient of ${\mathcal {L}}(w, b, \lambda )$ with respect to *w* to zero. We also set the partial derivative of ${\mathcal {L}}(w, b, \lambda )$ with respect to *b* to zero. Doing so yields:

11 $$\begin{aligned} \nabla _w {\mathcal {L}}(w, b, \lambda )&= w - \sum _{i=1}^{N}\lambda _i y_i x_i = 0 \qquad \implies \qquad w = \sum _{i=1}^{N} \lambda _i y_i x_i \end{aligned}$$

12 $$\begin{aligned} \frac{\partial {\mathcal {L}}(w,b,\lambda )}{\partial b}&= -\sum _{i=1}^{N} \lambda _i y_i = 0 \qquad \implies \qquad \sum _{i=1}^{N} \lambda _i y_i = 0 \end{aligned}$$

Substituting Eqs. (11) and (12) into Eq. (10):

13 $$\begin{aligned} {\mathcal {L}}(\lambda ) = \sum _{i=1}^{N}\lambda _i - \frac{1}{2} \sum _{i=1}^{N}\sum _{j=1}^{N}\lambda _i \lambda _j x_i x_j y_i y_j \end{aligned}$$

Note that Eq. (13) is a function of $\lambda$ only. We want to maximize Eq. (13) with respect to the Lagrangian multipliers, and also ensure that $\lambda _i, \lambda _j \ge 0 \quad \forall i, j$, while satisfying Eq. (12).

### QUBO formulation

In order to convert SVM training into a QUBO problem, we write Eq. (13) as a minimization problem:

14 $$\begin{aligned} \min _{\lambda } \ {\mathcal {L}}(\lambda )&= \frac{1}{2} \sum _{i=1}^{N} \sum _{j=1}^{N} \lambda _i \lambda _j x_i x_j y_i y_j - \sum _{i=1}^{N} \lambda _i \qquad \lambda _i, \lambda _j \ge 0 \quad \forall i, j \end{aligned}$$

This can be written in a matrix form as follows:

15 $$\begin{aligned} \min _{\lambda } {\mathcal {L}}(\lambda ) = \frac{1}{2} \lambda ^T (X X^T \odot Y Y^T) \lambda - \lambda ^T 1_N \qquad \lambda \ge 0_N \end{aligned}$$

where, $1_N$ and $0_N$ represent *N*-dimensional vectors of ones and zeros respectively, and $\odot$ is the element-wise multiplication operation.

We now reintroduce the *K*-dimensional precision vector $P = [p_1, p_2, \ldots , p_K]^T$ as described in the “Linear regression” section of this paper, but only allow positive powers of 2 in order to impose the non-negativity constraint on $\lambda$. We also introduce *K* binary variables ${\hat{\lambda }}_{ik}$ for each Lagrangian multiplier such that:

16 $$\begin{aligned} \lambda _i&= \sum _{k=1}^K p_k {\hat{\lambda }}_{ik} \qquad \forall i = 1, 2, \ldots , N \end{aligned}$$

where, $p_k$ denotes the $k{\text {th}}$ entry in the precision vector *P*. Next, we vertically stack all binary variables:

17 $$\begin{aligned} {\hat{\lambda }}&= [{\hat{\lambda }}_{11} \ \ldots \ {\hat{\lambda }}_{1K} \ {\hat{\lambda }}_{21} \ \ldots \ {\hat{\lambda }}_{2K} \ \ldots \ {\hat{\lambda }}_{N1} \ \ldots \ {\hat{\lambda }}_{NK} ]^T \end{aligned}$$

We now define the precision matrix as follows:

18 $$\begin{aligned} {\mathcal {P}} = I_N \otimes P^T \end{aligned}$$

Notice that:

19 $$\begin{aligned} \lambda&= {\mathcal {P}} {\hat{\lambda }} \end{aligned}$$

Finally, we substitute the value of $\lambda$ from Eq. (19) into Eq. (15):

20 $$\begin{aligned} \min _{{\hat{\lambda }} \in {\mathbb {B}}^{NK}} \ {\mathcal {L}} ({\hat{\lambda }}) = \frac{1}{2} {\hat{\lambda }}^T {\mathcal {P}}^T (X X^T \odot Y Y^T) {\mathcal {P}} {\hat{\lambda }} - {\hat{\lambda }}^T {\mathcal {P}}^T 1_N \end{aligned}$$

Equation (20) is identical to Eq. (1) with $z = {\hat{\lambda }}$, $A = \frac{1}{2}{\mathcal {P}}^T (X X^T \odot Y Y^T) {\mathcal {P}}$, $b = -{\mathcal {P}}^T 1_N$, and $M = KN$. Hence, we have converted the SVM training problem from Eq. (10) into a QUBO problem in Eq. (20), which can be solved on adiabatic quantum computers.

### Computational complexity

We begin our theoretical analysis by defining the space complexity with respect to the number of qubits needed to solve the QUBO. The SVM training problem stated in Eq. (15) contains ${\mathcal {O}}(N)$ variables ($\lambda$) and ${\mathcal {O}}(Nd)$ data (*X* and *Y*). The QUBO formulation of the SVM training problem stated in Eq. (20) consists of the same amount of data. However, as part of the QUBO formulation, we introduced *K* binary variables for each Lagrangian multiplier in the original problem (Eq. 15). So, the total number of variables in Eq. (20) is ${\mathcal {O}}(KN)$. So, the qubit footprint (or space complexity) of this formulation would be ${\mathcal {O}}(N^2 K^2)$ after embedding onto the hardware.

The time complexity of classical SVM algorithms is known to be ${\mathcal {O}}(N^3)$^46^. We analyze the time complexity for training an SVM model in three parts as outlined in “Linear regression” section. Firstly, the time complexity for converting Problem (9) into a QUBO problem can be inferred from Eqs. (14) and (16) as ${\mathcal {O}}(N^2K^2)$. Secondly, the time taken to embed the (*NK*)-sized QUBO problem on the quantum computer is ${\mathcal {O}}(N^2K^2)$ (see “Linear regression” section for more details). Lastly, for the reasons mentioned in the “Linear regression” section, it is not straight forward to get a realistic estimate of the time complexity of the quantum annealing process. However, a constant annealing time in conjunction with a constant number of repetitions seems to work well in practice on an adiabatic quantum computer of fixed and finite size as explained in “Regression” section. So, the total time complexity is ${\mathcal {O}}(N^2K^2)$.

Note that the qubit footprint ${\mathcal {O}}(N^2K^2)$ and time complexity ${\mathcal {O}}(N^2K^2)$ assume that *K* is a variable. If the precision for all parameters (${\hat{\lambda }}$) is fixed (e.g. limited to 32-bit or 64-bit precision), then *K* becomes a constant factor. The resulting qubit footprint would be ${\mathcal {O}}(N^2)$, and time complexity would also be be ${\mathcal {O}}(N^2)$. This time complexity is an order of magnitude better than the classical algorithm (${\mathcal {O}}(N^3)$).

## Balanced *k*-means clustering

### Background

**Figure 3:**
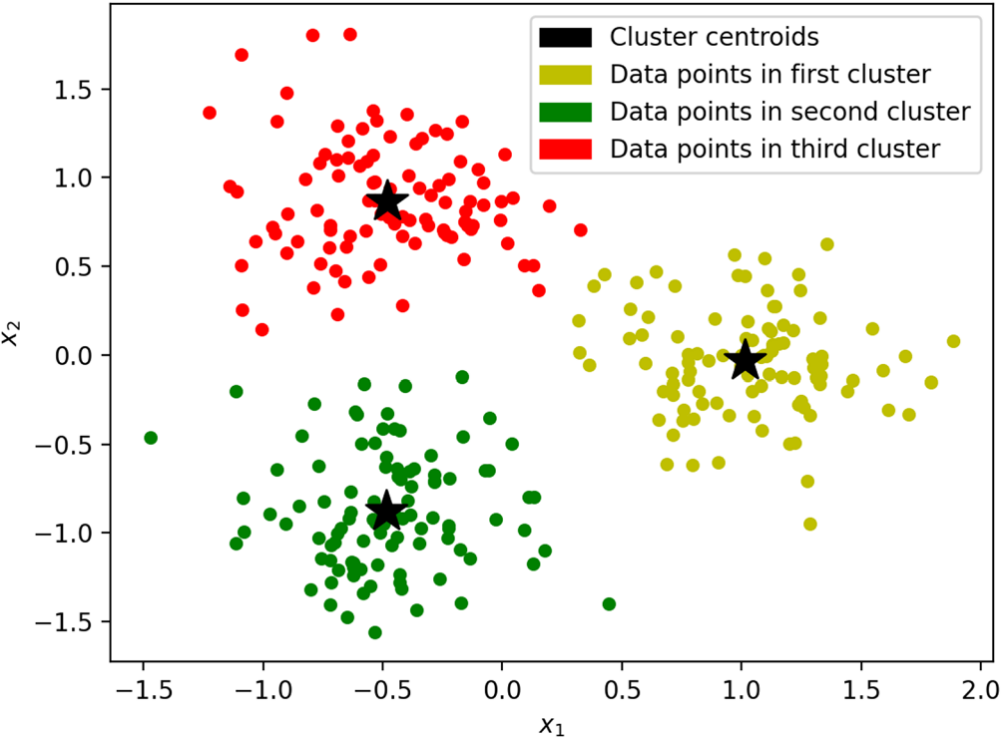
Training a balanced k-means clustering model ($k = 3$) on training data (yellow, green, and red dots).

*k*-Means clustering is an unsupervised machine learning model that partitions training data into *k* clusters such that each point belongs to the cluster with the nearest centroid. The optimal cluster assignments of the training data minimizes within-cluster variance. Balanced *k*-means clustering is a special case of the *k*-means model where each cluster contains approximately *N*/*k* points as shown in Fig. 3. Balanced clustering models have applications in a variety of domains including network design^47^, marketing^48^, and document clustering^49^.

Quantum approaches to training clustering models have been discussed in the literature. Ushijima-Mwesigwa et al. demonstrate partitioning a graph into *k* parts concurrently using quantum annealing on the D-Wave 2X machine^50^. Kumar et al. present a QUBO formulation for *k*-clustering that differs from the *k*-means model^51^. Bauckhage et al. propose a QUBO formulation for binary clustering ($k = 2$)^52^ and *k*-medoids clustering^53^. Our QUBO formulation for balanced *k*-means clustering synthesizes a number of ideas proposed in the literature.

Given training data $X \in {\mathbb {R}}^{N \times d}$, we would like to partition the *N* data points into *k* clusters $\Phi = \{\phi _1, ..., \phi _k\}$. Let the centroid of cluster $\phi _i$ be denoted as $\mu _i$. Formally, training the generic *k*-means clustering model is expressed as:

21 $$\begin{aligned} \min _{\Phi } \ \sum _{i = 1}^k \frac{1}{2|\phi _i|} \sum _{x, y \ \in \ \phi _i} || x - y ||^2 \end{aligned}$$

In the case that each cluster is of equal size, $|\phi _i|$ is constant, and Problem (21) reduces to:

22 $$\begin{aligned} \min _{\Phi } \ \sum _{i = 1}^k \sum _{x, y \ \in \ \phi _i} || x - y ||^2 \end{aligned}$$

Note that for most applications of balanced clustering, the cluster sizes are only approximately equal to one another. In these cases, the solution to Problem (22) may not be the exact solution to Problem (21). Classically, the *k*-means clustering problem is solved heuristically through an iterative approach known as Lloyd’s algorithm. A modified version of this algorithm is used for balanced *k*-means clustering to uphold the constraint that no cluster contains more than *N*/*k* points^54^. This modified version of Lloyd’s algorithm runs in ${\mathcal {O}}(N^{3.5} k^{3.5} )$ time on classical computers^55^.

### QUBO formulation

To formulate Problem (22) as a QUBO problem, it will be useful to define a matrix $D \in {\mathbb {R}}^{N \times N}$ where each element is given by:

23 $$\begin{aligned} d_{ij} = ||x_i - x_j||^2 \end{aligned}$$

where $x_i$ and $x_j$ are the $i{\text {th}}$ and $j{\text {th}}$ data points in *X*. We also define a binary matrix ${\hat{W}} \in {\mathbb {B}}^{N \times k}$ such that ${\hat{w}}_{ij} = 1$ if and only if point $x_i$ belongs to cluster $\phi _j$. Since we are assuming clusters of the same size, each column in ${\hat{W}}$ should have approximately *N*/*k* entries equal to 1. Additionally, since each data point belongs to exactly one cluster, each row in ${\hat{W}}$ must contain exactly one entry equal to 1. Using this notation, the inner sum in Problem (22) can be rewritten:

24 $$\begin{aligned} \sum _{x, y \in \phi _j} || x - y ||^2 = {\hat{w}}'^{T}_{j} D {\hat{w}}'_j \end{aligned}$$

where ${\hat{w}}'_j$ is the $j{\text {th}}$ column in ${\hat{W}}$. From this relation, we can cast Problem (22) into a constrained binary optimization problem. First, we vertically stack the *Nk* binary variables in ${\hat{W}}$ as follows:

25 $$\begin{aligned} {\hat{w}} = [{\hat{w}}_{11} \ \ldots \ {\hat{w}}_{N1} \ {\hat{w}}_{12} \ \ldots \ {\hat{w}}_{N2} \ \ldots \ {\hat{w}}_{1k} \ \ldots \ {\hat{w}}_{Nk}]^T \end{aligned}$$

Provided the constraints on ${\hat{w}}$ are upheld, Problem (22) is equivalent to:

26 $$\begin{aligned} \min _{{\hat{w}}} \ {\hat{w}}^T (I_k \otimes D) {\hat{w}} \end{aligned}$$

where $I_k$ is the *k*-dimensional identity matrix.

We can add the constraints on ${\hat{w}}$ by including penalty terms that are minimized when all conditions are satisfied. First, we account for the constraint that each cluster must contain approximately *N*/*k* points. For a given column ${\hat{w}}'_j$ in ${\hat{W}}$, this can be enforced by including a penalty of the form:

27 $$\begin{aligned} \alpha ({\hat{w}}{'}_j^T {\hat{w}}'_j - N/k)^2 \end{aligned}$$

where $\alpha$ is a constant factor intended to make the penalty large enough that the constraint is always upheld. Dropping the constant term $\alpha (N/k)^2$, this penalty is equivalent to ${\hat{w}}{'}_j^T \alpha F {\hat{w}}'_j$ where *F* is defined as:

28 $$\begin{aligned} F = 1_N - \frac{2N}{k} I_N \end{aligned}$$

Using this formulation, the sum of all column constraint penalties is:

29 $$\begin{aligned} {\hat{w}}^T (I_k \otimes \alpha F ) {\hat{w}} \end{aligned}$$

Next, we account for the constraint that each point belongs to exactly 1 cluster. For a given row ${\hat{w}}_i$, this can be enforced by including a penalty of the form:

30 $$\begin{aligned} \beta ({\hat{w}}_i^T {\hat{w}}_i - 1)^2 \end{aligned}$$

where $\beta$ is a constant with the same purpose as $\alpha$ in Eq. (27). Dropping the constant term, this penalty is equivalent to ${\hat{w}}_i^T \beta G {\hat{w}}_i$ where *G* is defined as:

31 $$\begin{aligned} G = 1_k - 2 I_k \end{aligned}$$

To find the sum of all row constraint penalties, we first convert the binary vector ${\hat{w}}$ into the form ${\hat{v}}$ shown below:

32 $$\begin{aligned} {\hat{v}} = [w_{11} \ldots w_{1k} \ w_{21} \ldots w_{2k} \ldots w_{N1} \ldots w_{Nk}]^T \end{aligned}$$

This can be accomplished through a linear transformation $Q {\hat{w}}$ where each element in $Q \in {\mathbb {B}}^{Nk \times Nk}$ is defined as:

33 $$\begin{aligned} q_{ij} = {\left\{ \begin{array}{ll} 1 &{} j = N {\text {mod}}(i - 1, k) + \lfloor \frac{i - 1}{k} \rfloor + 1 \\ 0 &{} \text {else} \\ \end{array}\right. } \end{aligned}$$

After the transformation, the sum of all row constraint penalties is given by ${\hat{v}}^T (I_N \otimes \beta G) {\hat{v}}$. This can be equivalently expressed as:

34 $$\begin{aligned} {\hat{w}}^T Q^T (I_N \otimes \beta G) Q {\hat{w}} \end{aligned}$$

Combining the penalties from Eqs. (29) and (34) with the constrained binary optimization problem from Eq. (26), Problem (22) can be rewritten as:

35 $$\begin{aligned} \min _{{\hat{w}}} \ {\hat{w}}^T (I_k \otimes (D + \alpha F) + Q^T (I_N \otimes \beta G) Q) {\hat{w}} \end{aligned}$$

Equation (35) is identical to Eq. (1) with $z = {\hat{w}}$, $A = (I_k \otimes (D + \alpha F) + Q^T (I_N \otimes \beta G) Q)$, and $b = 0$. Thus, we have converted Eq. (22) into a QUBO problem which can be solved on adiabatic quantum computers.

### Computational complexity

The balanced *k*-means clustering problem stated in Eq. (22) contains ${\mathcal {O}}(Nd)$ data and ${\mathcal {O}}(N)$ variables. In our QUBO formulation, we introduce *k* binary variables for each variable in the original problem. Thus, the total number of variables in Eq. (35) is ${\mathcal {O}}(Nk)$. This translates to a quadratic qubit footprint of ${\mathcal {O}}(N^2 k^2)$.

While an exact solution to the generic *k*-means clustering model (Problem 21) requires ${\mathcal {O}}(N^{kd + 1})$ time^56^, a classical algorithm for balanced *k*-means clustering will converge to a locally optimal solution in ${\mathcal {O}}(N^{3.5} k^{3.5} )$ time^55^. To compute the time complexity for converting Eq. (22) into a QUBO problem, we can rewrite Eq. (35) as follows:

36 $$\begin{aligned} \min _{W} \sum _{l = 1}^k \sum _{j = 1}^N \sum _{i = 1}^N \sum _{m = 1}^d w_{il} (x_{im} - x_{jm})^2 w_{jl} + \alpha \sum _{l = 1}^k \sum _{j = 1}^N \sum _{i = 1}^N w_{il} f_{ij} w_{jl} + \beta \sum _{l = 1}^N \sum _{j = 1}^k \sum _{i = 1}^k w_{li} g_{ij} w_{lj} \end{aligned}$$

From Eq. (36), the time complexity is ${\mathcal {O}}(N^2 k d)$, which is dominated by the first term. Embedding a QUBO problem having ${\mathcal {O}}(Nk)$ variables takes ${\mathcal {O}}(N^2 k^2)$ time using the embedding algorithm proposed by Date et al.^5^. For the reasons mentioned in the “Linear regression” section, it is not straight forward to get a realistic estimate of the time complexity of the quantum annealing process. However, a constant annealing time in conjunction with a constant number of repetitions seems to work well in practice on an adiabatic quantum computer of fixed and finite size as explained in the “Linear regression” section. Therefore, the total time complexity for the quantum algorithm is ${\mathcal {O}}(N^2 k (d + k))$. This time complexity is better than the worst case time complexity of the classical algorithm $({\mathcal {O}}(N^{3.5} k^{3.5}))$. However, the number of iterations in the classical algorithm varies greatly depending on the quality of the initial guess at the cluster centroids. In some cases, the classical algorithm may converge in much less than ${\mathcal {O}}(N^{3.5} k^{3.5})$ time and outperform its quantum counterpart.

## Conclusion

As the task of training machine learning models becomes more computationally intensive, devising new methods for efficient training has become a crucial pursuit in machine learning. The process of training a given model can often be formulated as a problem of minimizing a well-defined error function for a given machine learning model. Given the power of quantum computers to approximately solve certain hard optimization problems with great efficiency as well as the demonstration of quantum supremacy by Google, we believe quantum computers can accelerate training of machine learning models. In this paper, we posed the training problems for three machine learning models (linear regression, support vector machine, and balanced *k*-means clustering) as QUBO problems to be solved on adiabatic quantum computers like D-Wave 2000Q. Furthermore, we analyzed the associated time and space complexity of our formulations and provided a theoretical comparison to the state-of-the-art classical methods for training these models. Our results are promising for training machine learning models on quantum computers in the future.

In the future, we would like to empirically evaluate the performance of our quantum approaches on real quantum computers. We would also like to compare the performance of our quantum approaches to state-of-the-art classical approaches. Finally, we would like to formulate other machine learning models such as logistic regression, restricted Boltzmann machines, deep belief networks, Bayesian learning and deep learning as QUBO problems that could potentially be trained on adiabatic quantum computers.
